# Impact of Maternal and Neonatal Factors on Thyroid-Stimulating Hormone Concentrations in Newborns in Eastern Morocco

**DOI:** 10.7759/cureus.85964

**Published:** 2025-06-13

**Authors:** Fatima Wahoud, Rim Amrani

**Affiliations:** 1 Maternal Child and Mental Health Research Laboratory, Mohammed First University, Faculty of Medicine and Pharmacy, Oujda, MAR

**Keywords:** congenital hypothyroidism, eastern morocco, neonatal screening, newborn, tsh levels

## Abstract

Introduction: Congenital hypothyroidism (CH) is a major preventable cause of intellectual disability. Neonatal screening based on thyroid-stimulating hormone (TSH) levels enables early detection and intervention. While maternal health conditions and exposures during pregnancy are known to affect newborn TSH levels, the newborn's own characteristics may also influence these results.

Methods: A cross-sectional study was conducted on 1,119 mothers and their newborns in the region of Eastern Morocco. A survey was conducted to collect sociodemographic data, anthropometric data, and medical history. For the newborns, in addition to a clinical examination, heel prick samples were collected on blotting papers, and an immunofluorimetric method was used to quantify TSH.

Results: The analysis of maternal factors revealed a highly significant association between maternal thyroid dysfunction and TSH concentrations in newborns (p < 0.001, OR = 11.365). Drug use during pregnancy was also strongly associated (p = 0.014, OR = 3.230), especially anti-thyroid treatment (p < 0.001, OR = 18.218). Conversely, other factors, such as anthropometric indicators and pregnancy-related conditions (hypertension, diabetes), showed no statistically significant association. As for neonatal factors, high TSH levels were significantly more frequent in preterm infants (p = 0.006, OR = 4.09), whereas no significant associations were found with other factors (sex, birth weight, or length). Hypotonia and feeding difficulties were more prevalent in newborns with elevated TSH but did not reach statistical significance.

Conclusion: Both maternal and neonatal factors may influence neonatal TSH values, in particular, prematurity. The results also show the direct impact of maternal thyroid disorders on the newborn’s thyroid function. This explains the importance of contextual interpretation of TSH results in neonatal screening, but also the importance of screening and treating maternal thyroid dysfunction during pregnancy.

## Introduction

Congenital hypothyroidism (CH) is the most common cause of preventable intellectual disability in children, with a global incidence ranging between 1:2,500 and 1:4,000 live births. In Morocco, studies conducted between 1996 and 2003 reported a comparatively higher incidence, ranging from 1:1,138 to 1:1,952 live births, highlighting a potential public health concern [1]. Despite the high prevalence, clinical identification of CH at birth is often challenging due to the absence of overt signs in the neonatal period. However, early initiation of treatment is critical, as delays can lead to irreversible neurodevelopmental damage [2].

The majority of infants with CH are diagnosed based on elevated levels of thyroid-stimulating hormone (TSH) detected during routine neonatal screening. This screening is typically performed using dried blood spot samples collected shortly after birth [3]. In response to the proven effectiveness of early detection, numerous countries have adopted national neonatal screening programs aimed at identifying CH before the onset of clinical symptoms [4,5].

TSH concentration in the neonatal period is primarily used as a marker of thyroid function, but it is increasingly recognized that TSH levels may be influenced by a multitude of non-thyroidal factors. These include physiological, maternal, obstetric, and neonatal variables, some of which may confound the screening results or lead to unnecessary recalls or misclassifications [6].

Indeed, previous research has shown that maternal thyroid disorders, medication use during pregnancy, delivery complications, and neonatal factors such as prematurity, birth weight, and sex can influence neonatal TSH concentrations [7]. However, the evidence remains heterogeneous and, at times, contradictory. Many studies have been limited by small sample sizes or the lack of control for confounding variables, making it difficult to draw definitive conclusions about the exact role each factor plays in determining neonatal TSH levels [8].

In Morocco, although neonatal CH screening has been gradually implemented, regional data exploring the maternal and neonatal determinants of TSH variability remain scarce. In particular, the Oriental region of the country, characterized by sociodemographic and healthcare access disparities, has not been comprehensively studied. According to the regional health department, although specific data on prevalence are limited, several factors suggest that CH is under-detected. Understanding the local patterns and predictors of elevated TSH in this population is essential to optimize screening strategies and ensure timely treatment.

## Materials and methods

Objective

This study aimed to evaluate the association between maternal and neonatal factors and neonatal TSH concentrations among newborns in the region of Eastern Morocco.

Design and implementation

This study is part of an ongoing cross-sectional descriptive project conducted across the region of Eastern Morocco. A total of 1,119 mothers and their newborns were recruited between March 2024 and January 2025 from maternity wards in eight provinces: Oujda, Nador, Berkane, Guercif, Taourirt, Jerada, Driouch, and Figuig.

A non-probability convenience sampling method was used to recruit participants. All mothers who provided informed consent and whose newborns were screened after 24 hours of life were included in the study. However, newborns who were screened before 24 hours of life (whose peak TSH is physiologically increased) or whose clinical or questionnaire data were incomplete were excluded from the study.

Data collection followed a retrospective-prospective approach. Sociodemographic information, including place of residence, maternal age, consanguinity, and medical history, was collected using a structured questionnaire, which was pretested to ensure clarity and adapted from a tool previously validated in perinatal studies. Mothers were asked about the progression of their pregnancy and the circumstances of childbirth. Weight and height were measured for all mothers using standardized procedures. After delivery, the newborn’s sex, birth weight, and gestational age were also recorded.

To assess TSH concentration levels, dried capillary blood samples were collected via heel prick between the second and the fifth day after birth and analyzed using the Auto DELFIA Neonatal hTSH kit (Revvity Inc., Waltham, MA). Quantification of TSH was performed using a time-resolved immunofluorometric assay. TSH concentrations below 15 µU/mL were considered within the normal range in accordance with national standards.

To ensure inter-site consistency across the eight participating provinces, all healthcare personnel involved in data collection received standardized training prior to the start of the study.

Ethics

This study was approved by the Biomedical Research Ethics Committee of Oujda (approval No. 04/2023). Informed written consent was obtained from all participating mothers prior to inclusion. Data confidentiality and participant anonymity were strictly maintained. Neonatal blood samples were procured in accordance with national and international ethical standards.

Statistical analyses

All data were entered and analyzed using SPSS software, version 25 (IBM Corp., Armonk, NY). Descriptive statistics were used to summarize maternal and neonatal characteristics. Data quality was reviewed before analysis. Incomplete records or missing questionnaire data were excluded from the final dataset. Odds ratios (OR) and their 95% confidence intervals (CI) were calculated to assess the strength of association between categorical variables in bivariate analyses.

The Kolmogorov-Smirnov test was applied to assess the normality of continuous variables. As the distribution of most quantitative variables was non-normal, they were presented as medians and ranges.

Qualitative variables were compared using Pearson's chi-square test or Fisher’s exact test when expected cell counts were below 5. Quantitative variables were compared using the Mann-Whitney U test (for two groups) or the Kruskal-Wallis test (for more than two groups).

A p-value < 0.05 was considered statistically significant.

## Results

Characteristics of study participants

A total of 1,119 mother-newborn pairs from eight provinces were recruited in the study: Oujda (38.16%), Nador (17.07%), Berkane (12.33%), Jerada (11.17%), Guercif (6.34%), Taourirt (6.17%), Figuig (5.09%), and Driouch (3.66%). The majority of mothers were from an urban area (70.33%) versus 29.66% from suburban or rural areas. As for the inbreeding, the rate declared was 19.75%. First- and second-degree consanguinity were observed in 7.86% and 11.89% of the population, respectively. The province of Figuig had the highest rate (33.33%; p<0.001), and consanguinity was two times higher in rural areas than urban areas (31.52% vs 15.75%).

The median age of mothers was 30 (16-47) years, and 87.04% of them were aged from 20 to 39. Nearly half of the mothers (49.82%) were classified as overweight, with a body mass index (BMI) between 25 kg/m² and 29.9 kg/m², while over 22% were obese, presenting a BMI above 30 kg/m². Approximately 17.34% of women declared that they were suffering from some pathologies during pregnancy, such as high blood pressure (31.44%), diabetes (24.23%), and thyroid disorders (15.98%). Along with this, 38.70% of mothers used medications: iron (66.51%), anti-hypertensives (7.39%), anti-diabetics (2.08%), anti-thyroid (4.85%), and only 5.77% of women consumed iodine. The consumption of medicinal plants (undefined nature) was declared by 3.23% of the women interviewed.

For the newborn population, males represented 51.12% against 48.88% females, corresponding to a male-to-female ratio of 1.05. In total, 88.8% of newborns were at term with a median birth weight of 3400 (1850-5250) g. It is worth noting that newborns with a weight ≥2500 g represented the majority (93.74%), with no significant difference between the two genders (93.24% in girls and 94.23% in boys).

**Figure 1:**
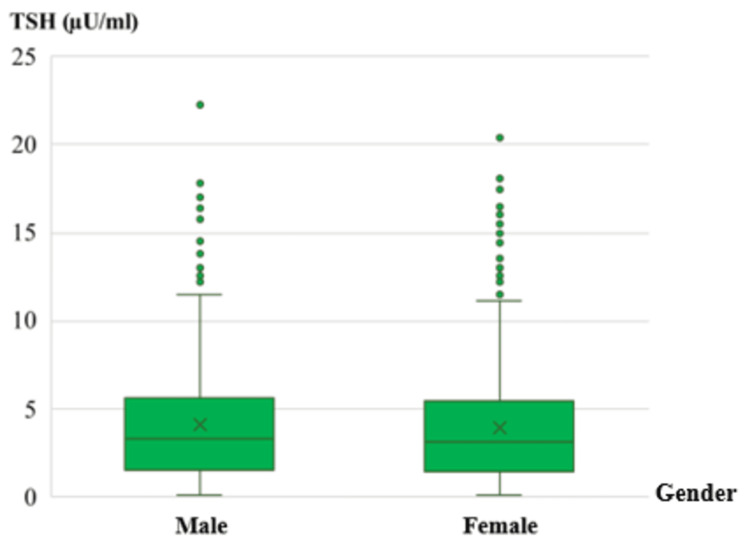
TSH concentration levels according to newborns gender

As for screening results, analysis of TSH concentration showed that the median TSH level was similar in boys (3.3 µU/ml (0.1-22.30)) and girls (3.1 µU/ml [0.1-20.40]). The majority of newborns (98.39%) had TSH concentrations ≤15 µU/ml, in accordance with normal values. While 18 newborns (1.61%) had TSH > 15 µU/ml, the variation in TSH concentrations between male and female newborns was not statistically significant (p = 0.295) (Figure 1).

Study of associations

According to maternal factors, there was no association between TSH levels in newborns and some mothers' demographic factors, including age, place of residence, and consanguinity. However, notably high levels of TSH were observed, particularly in Figuig (p = 0.024, OR = 3.878), contrary to the other provinces. Unexpectedly, when mothers were overweight with a BMI between 25 and 29.9 kg/m², newborns were less likely to have high levels of TSH concentration (p=0.034; OR=0.283).

The evaluation of the effect of the mother's medical history on TSH levels in the newborn shows a statistically significant association in cases of maternal thyroid dysfunction. In fact, those with thyroid disorders during pregnancy showed an increased risk of having newborns with TSH levels >15 µU/mL (p < 0.001, OR = 11.365). Along with this, the use of medication during pregnancy was also strongly associated (p = 0.014, OR = 3.230), especially anti-thyroid (p < 0.001, OR = 18.218). Unexpectedly, the use of medicinal plants, although rare, showed a significant association with elevated TSH concentrations (p<0.001, OR=11.344). Otherwise, other maternal factors such as hypertension, diabetes, or lack of iodine supplementation did not show a statistically significant association with TSH concentrations in newborns (Table 1).

**Table 1:** Maternal risk factors associated with neonatal TSH levels. * relative risk. Values are calculated from bivariate logistic regression. Abbreviations: TSH, thyroid-stimulating hormone; BMI, body mass index; NS, not significant; OR: odds ratio, CI, confidence interval

Settings	TSH ≤ 15 (µU/ml)	TSH > 15 (µU/ml)	Total	p-value	OR (IC 95%)
Number	1101 (98.39%)	18 (1.61%)	1119 (100%)	-	-
BMI (kg/m²)					
25-29.9 (overweight)	553 (50.23%)	4 (22.22%)	557 (49.82%)	0.034	0.283 (0.093-0.866)
Provinces					
Figuig	54 (4.90%)	3 (16.67%)	57 (5.09%)	0.024	3.878 (1.090-13.800)
Pathologies	190 (17.26%)	4 (22.22%)	194 (17.34%)	NS	1.370 (0.446-4.208)
Thyroid dysfunction	27 (14.21%)	4 (100%)	31 (15.98%)	<0.001	11.365 (3.510-36.801)
Drug use	421 (38.24%)	12 (66.67%)	433 (38.70%)	0.014	3.230 (1.203-8.672)
Anti-thyroid	17 (4.04%)	4 (33.33%)	21 (4.85%)	<0.001	18.218 (5.432-61.10)
Iodine supplements	25 (5.94%)	0 (0%)	25 (5.77%)	NS	0.984 (0.976-0.991)*
Medicinal plants	12 (2.85%)	2 (16.67%)	14 (3.23%)	<0.001	11.344 (2.345-54.866)

Regarding neonatal characteristics, the study showed that some factors had an effect on the levels of TSH concentration. Notably, preterm newborns were found at significantly higher risk of having TSH concentrations above 15 µU/mL (p = 0.006, OR = 4.09). However, neonatal sex, weight, and length were not significantly associated with TSH elevation (p > 0.05). Additionally, although clinical signs such as feeding difficulties, hypotonia, and prolonged jaundice were more frequently observed among newborns with elevated TSH, these associations did not reach statistical significance (Table 2).

**Table 2:** Neonatal risk factors associated with neonatal TSH levels. * relative risk. Values are calculated from bivariate logistic regression. Abbreviations: TSH, thyroid-stimulating hormone; NS, not significant; OR, odds ratio; CI, confidence interval

Neonatal characteristic	TSH>15 (µU/ml)	TSH ≤15 (µU/ml)	p-value	Odds ratio (95% CI)
Prematurity	27.78%	7.48%	0.006	4.09 (1.49–11.23)
Male sex	55.56%	51.05%	0.894	1.07 (0.41–2.80)
Birth weight < 2500 g	16.67%	10.16%	0.686	1.27 (0.40–4.04)
Length < 47 cm	22.22%	20.17%	0.911	0.94 (0.32–2.74)
Feeding difficulties	22.22%	12.29%	>0.05*	NS
Hypotonia	16.67%	11.65%	>0.05*	NS
Prolonged jaundice	11.11%	5.80%	>0.05*	NS

## Discussion

The development of the thyroid gland in newborns is sensitive to maternal factors during pregnancy and delivery [9]. Out of 1,119 screenings, 18 newborns had TSH levels above 15 µU/mL, which means a recall rate of 1.6%. The thresholds used in different screening programs can directly influence the incidence rate of CH [9]. This has been demonstrated in several countries, including Greece, where the incidence of CH was 1:1758 and 1:2441 with TSH thresholds of 10 and 20 mU/L, respectively [10, 11].

As for the socio-demographic characteristics, 12.96% of the mothers were aged below 20 or above 39 years. But this had no association with neonatal TSH levels. According to Luo et al. (2020), the risk of permanent CH in newborns of mothers aged over 40 years is significantly higher than that of younger mothers (aged between 20 and 30 years), whereas maternal age under 20 years is a protective factor against CH [9].

Despite the high rate of obesity in women (over 22%), TSH concentration was normal in all newborns of mothers with a BMI ≥30 kg/m². But the newborns of mothers with a BMI inferior to 30 kg/m² were protected against an elevated level of TSH concentration (OR: 0.283; 95% CI (0.093-0.866); p=0.034). In previous research about the determinants of TSH in umbilical cord blood, the results were the same [7]. Interestingly, our analysis revealed a lower risk of elevated TSH concentrations among newborns of overweight mothers. While this association was unexpected, several hypotheses may be considered. Maternal overweight could influence fetal thyroid function through altered maternal thyroid hormone levels, inflammatory status, or placental physiology. Alternatively, this finding may reflect the influence of unmeasured confounding factors, such as iodine intake, dietary patterns, or differences in prenatal care access and quality among overweight women. Although the current study was not designed to investigate these mechanisms in depth, this result highlights the need for further research to better understand the biological and contextual factors involved.

As for the women's place of residence, the distribution of the different provinces of the region shows a predominance of women from Oujda (38.15%) because of the accessibility to the screening centres. The small proportion (5.09%) of women from Figuig reported a consanguineous marriage in 33.33% of cases. In this context, the risk of neonatal TSH above 15µU/mL was three times higher than in the other provinces (OR: 3.878; 95% CI (1.090-13.800); p=0.024). It is possible that high rates of consanguineous marriage, with an associated risk of hereditary thyroid hormone deficiency, and ethnicity are important factors in the increased incidence of CH [12]. 

Talking about pathologies during pregnancy, the mothers with thyroid disorders showed an increased risk of having newborns with TSH levels >15 µU/mL (OR: 11.365; 95% CI (3.510-36.801); p < 0.001), hence the importance of screening and treating these pathologies in mothers during pregnancy. Although the history of thyroid disease in the mother is generally considered to be a risk factor for CH, few studies have examined its direct effect on neonatal TSH levels [13].

A strong association was found for medication taken during pregnancy (OR: 3.230; 95% CI (1.203-8.672); p = 0.014), especially antithyroid (Levothyrox) (OR: 18.218; 95% CI (5.432-61.10); p < 0.001), in contrast to iodine supplementation. Because Morocco is one of the countries with an adequate iodine intake, the most frequent cause of CH is maternal treatment with antithyroid [14]. Moreover, the use of neonatal screening TSH in the estimation of maternal iodine has been questioned due to confounding factors (time and type of sampling, maternal iodine status, and exposure to antiseptics containing iodine). Unexpectedly, despite the rare use of medicinal plants, there was a significant association between it and the elevated TSH concentrations (OR: 11.344; 95% CI (2.345-54.866); p<0.001). This result should be interpreted with caution. The study did not capture the specific types of plants, their dosages, or preparation methods. Therefore, this finding remains exploratory and highlights the need for further research to identify the specific herbal agents involved and their potential impact on neonatal thyroid function. However, as no randomised clinical trials or ongoing studies were found regarding the effects of medicinal plants in CH, no effect was established [15]. 

On the other hand, the results revealed that among the different neonatal variables, prematurity was significantly associated with elevated TSH concentrations (>15 µU/mL). This finding is compatible with other research indicating that premature infants are more likely to have transient hypothyroidism due to the immaturity of the hypothalamic-pituitary-thyroid (HPT) axis [16]. The immature feedback mechanisms and reduced thyroid hormone production in preterm newborns can lead to elevated TSH levels during the first days of life, which may hide a true congenital hypothyroidism [17]. Therefore, in clinical practice, it is recommended that the TSH screening be repeated in preterm infants or interpreted using gestational age-specific thresholds to avoid both false positives and negatives.

In contrast, other neonatal characteristics such as sex, birth weight, and length were not significantly associated with elevated TSH levels in our cohort. Although some studies have suggested minor sex-related differences in TSH levels, often reporting slightly higher values in males, our findings align with other studies that found no clinically meaningful difference between the two sexes [18]. Likewise, birth weight and length have occasionally been linked to TSH variability, particularly in the context of intra-uterine growth restriction or neonatal stress. However, after adjusting for prematurity, these anthropometric variables appear to have minimal influence on neonatal thyroid function [19].

The study also explored clinical signs typically associated with hypothyroidism, such as feeding difficulties, hypotonia, and prolonged jaundice. Although these symptoms were more frequent in newborns with elevated TSH, the associations did not reach statistical significance. This supports the idea that congenital hypothyroidism often goes asymptomatic in the neonatal period, which highlights the importance of universal biochemical screening rather than relying on symptoms [20]. These findings are essential for reinforcing screening guidelines, especially in settings with limited resources where clinical suspicion may not be sufficient for early diagnosis.

This study has some limitations, including its cross-sectional design, which prevents causal interpretation, and the use of convenience sampling, which may limit generalizability. Additionally, no multivariate analysis was conducted to control for confounding variables. The absence of long-term follow-up also prevents differentiation between transient and permanent forms of CH. However, the study also presents important strengths: a large and diverse sample from all provinces of Eastern Morocco, standardized data collection procedures, and a combined analysis of maternal and neonatal factors. As the first study of its kind in this region, it provides valuable insights to support improved neonatal screening strategies.

## Conclusions

This study highlights the potential influence of maternal and neonatal factors on neonatal TSH concentrations within the context of CH screening in Eastern Morocco. By applying a refined analytical approach, including multivariate analysis, we identified several significant associations while acknowledging the observational nature of the study. Although causality cannot be established, these findings contribute to a better understanding of contextual variables that may affect screening outcomes. Further prospective research, with long-term follow-up and detailed clinical data, is needed to validate these associations and support more targeted public health interventions.
